# Supplementary material for: An open-source framework for large-scale, flexible evaluation of biomedical text mining systems
Source: J Biomed Discov Collab. 2008 Jan 29;3:1. doi: 10.1186/1747-5333-3-1 (PMC2276192; doi:10.1186/1747-5333-3-1)
Supplement: Additional file 1 — Evaluation platform source code. The additional file contains the software evaluation framework discussed in this paper. The most current release can be downloaded from SourceForge.net [1]. See the accompanying README file for instructions on installation and use. Also included in the distribution is a collection of UIMA wrappers for some commonly used BioNLP tools and annotated corpora. [file 1747-5333-3-1-S1.zip › bionlp_uima_repository-1.0.1/docs/api/src-html/edu/uchsc/ccp/util/nlp/annotation/Annotation_Util.html]

```
001    /*
002     * Created on Aug 19, 2005
003     *
004     * TODO To change the template for this generated file go to
005     * Window - Preferences - Java - Code Style - Code Templates
006     */
007    package edu.uchsc.ccp.util.nlp.annotation;
008    
009    import java.util.ArrayList;
010    import java.util.List;
011    
012    import edu.uchsc.ccp.util.nlp.mention.ClassMention;
013    import edu.uchsc.ccp.util.nlp.mention.ClassMentionTypes;
014    import edu.uchsc.ccp.util.nlp.mention.SlotMention;
015    import edu.uchsc.ccp.util.nlp.mention.SlotMentionTypes;
016    
017    /**
018     * @author williamb
019     * 
020     */
021    public class Annotation_Util {
022    
023        public static ClassMention createTokenMention(String posLabel, String stem, String lemma, int tokenNumber) {
024            ClassMention cm = new ClassMention(ClassMentionTypes.TOKEN);
025            SlotMention sm;
026            if (posLabel != null) {
027                /* create POS slot */
028                sm = new SlotMention(SlotMentionTypes.TOKEN_PARTOFSPEECH);
029                sm.addSlotValue(posLabel);
030                cm.addSlotMention(sm);
031            }
032            if (stem != null) {
033                /* create stem slot */
034                sm = new SlotMention(SlotMentionTypes.TOKEN_STEM);
035                sm.addSlotValue(stem);
036                cm.addSlotMention(sm);
037            }
038            if (lemma != null) {
039                /* create lemma slot */
040                sm = new SlotMention(SlotMentionTypes.TOKEN_LEMMA);
041                sm.addSlotValue(lemma);
042                cm.addSlotMention(sm);
043            }
044            if (Integer.valueOf(tokenNumber) != null) {
045                /* create tokenNumber slot */
046                sm = new SlotMention(SlotMentionTypes.TOKEN_NUMBER);
047                sm.addSlotValue(Integer.toString(tokenNumber));
048                cm.addSlotMention(sm);
049            }
050            return cm;
051        }
052    
053        
054        public static void swapAnnotationInfo(TextAnnotation fromTA, TextAnnotation toTA) {
055            toTA.setAnnotationID(fromTA.getAnnotationID());
056            
057            toTA.setAnnotationSets(fromTA.getAnnotationSets());
058            toTA.setAnnotator(fromTA.getAnnotator());
059            toTA.setCoveredText(fromTA.getCoveredText());
060            toTA.setDocumentCollectionID(fromTA.getDocumentCollectionID());
061            toTA.setDocumentID(fromTA.getDocumentID());
062            toTA.setDocumentSectionID(fromTA.getDocumentSectionID());
063            for (Span span : fromTA.getSpans()) {
064                toTA.addSpan(span.clone());
065            }
066            
067        }
068        
069        public static List<Object> getSlotValuesByName(TextAnnotation ta, String slotName) {
070            List<SlotMention> slotMentions =  ta.getClassMention().getSlotMentionsByName(slotName);
071            List<Object> slotValues = new ArrayList<Object>();
072            for (SlotMention sm : slotMentions) {
073                slotValues.addAll(sm.getSlotValues());
074            }
075            return slotValues;
076        }
077        
078    }
```
